# Supplementary material for: Simultaneous Determination of Twenty Mycotoxins in the Korean Soybean Paste Doenjang by LC-MS/MS with Immunoaffinity Cleanup
Source: Toxins (Basel). 2019 Oct 12;11(10):594. doi: 10.3390/toxins11100594 (PMC6832528; doi:10.3390/toxins11100594)
Supplement: Supplementary file 1 [file toxins-11-00594-s001.pdf]

Supplementary Materials: Simultaneous Determination of Twenty Mycotoxins in the Korean Soybean Paste Doenjang by LC-MS/MS with Immunoaffinity Cleanup

So Young Woo, So Young Ryu, Fei Tian, Sang Yoo Lee, Su Been Park and Hyang Sook Chun

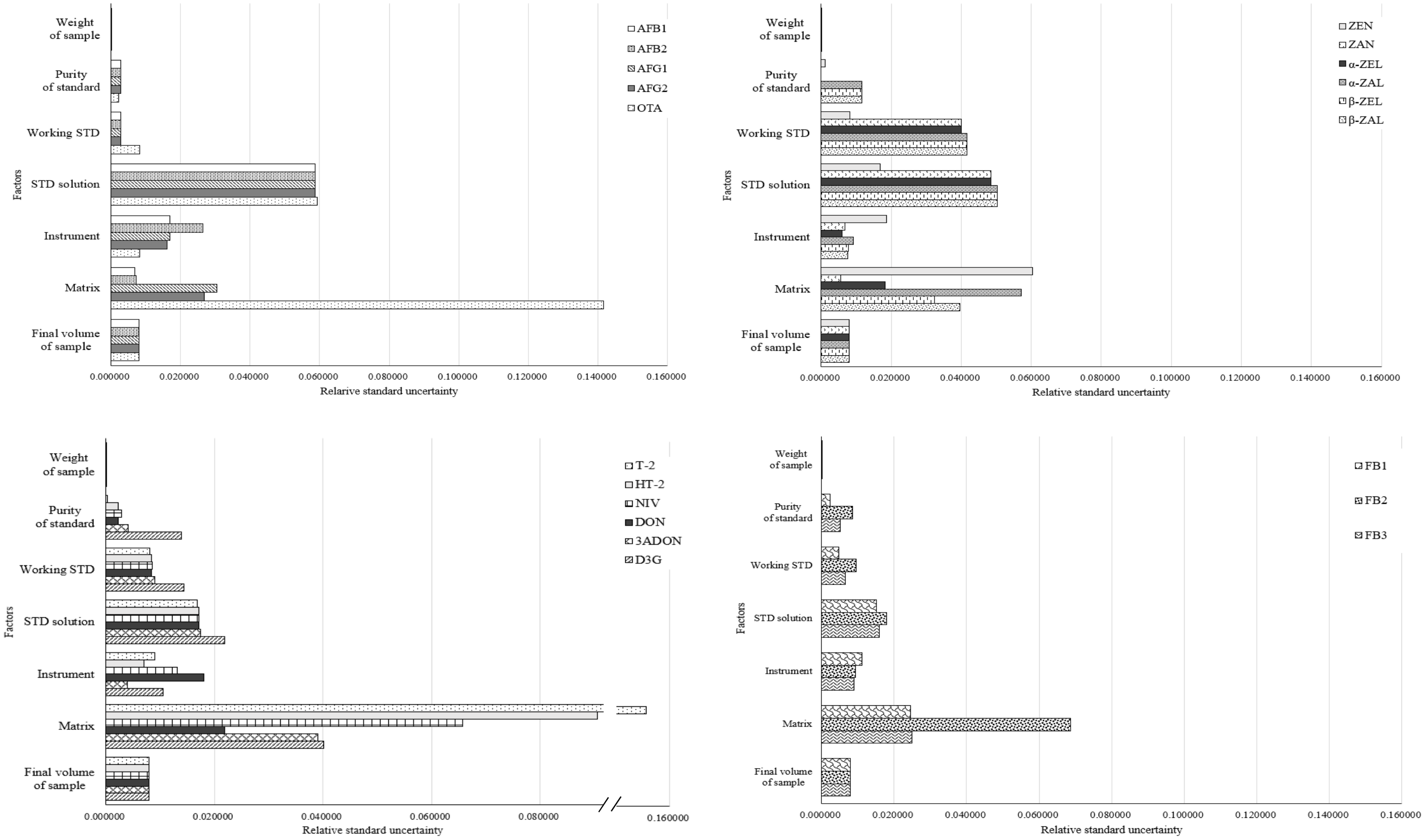

Figure S1. Contributions of each of the factors to the total uncertainty (10 μg/kg for aflatoxins (AFs) and ochratoxin A (OTA), 15 μg/kg for other mycotoxins).
